# A New Theory of Menstruation

**Published:** 1887-04

**Authors:** 


### *A NEW THEORY OF MENSTRUATION.*

In the "Presidential Address on Some Pending Questions in Gynæcology," delivered before the British Gynæcological Society (*British Medical Journal*, Jan. 22, 1887,) on January 12th, Mr. Lawson Tait calls attention to the subject of menstruation and refers to two papers read before the Society during the past year on this subject, in which a new theory of menstruation is presented by two different observers working upon different materials, in different ways, and approaching their subject from altogether different standpoints, and without the faintest kind of association, yet each arriving at conclusions absolutely identical. The authors of these two papers are Mr. Bland Sutton, of Liverpool, and Dr. Arthur Johnstone, of Danville, Ky.

It is well known that new theories of menstruation have been attributed to Mr. Tait, but Mr. Tait affirms that all he pleads for is a wholesome skepticism. He does not hold to the opinion that ovulation and menstruation stand as cause and effect.

The conclusion reached by Mr. Bland Sutton is that Macaque monkeys and baboons suffer a periodical loss of blood from the uterus. Unlike the human female, in them there is no shedding of the epithelial lining of the mucous membrane of the uterus and utricular glands. The amount of blood which escapes is very small in quantity.

Dr. Johnstone's paper, of which Mr. Tait says, "I am specially proud of it, because he is a typical example of the splendid race which the implantation of Britons on a new soil has produced, and I have had the privilege of ranking him among my pupils," is equally conclusive.

Dr. Johnstone advances the theory that menstruation is the result of a glandular function, and he shows that the menstrual organ is the endometrium. According to Dr. Johnstone's observations, a section of the uterus in a girl eleven years of age shows a mere coating of the columnar epithelium, without corpuscular development. A section of the endometrium of a girl aged thirteen, who had menstruated twice, shows more elaborately developed columnar epithelium, and the beginning of a corpuscular layer. The menstruating uterus of a woman of twenty shows abundant corpuscular development, constituting a thick endometrium, with its endometrium in process of casting, whilst a senile uterus at sixty shows merely the skeleton of the endometric structure, with almost complete exhaustion of its corpuscular elements and a total absence of epithelium. Both Mr. Sutton and Dr. Johnstone agree that in menstruation the epithelium of the tubes is not shed.

Mr. Tait argues that from these facts we have the explanation of a long series of most intricate physiological and pathological difficulties, the riddles of which seemed to be wholly beyond our grasp. "First," he says, "we have an explanation of the familiar fact that impregnation and menstruation seem to have a clear relation as to coincidence, the plain fact being that an impregnated ovum adheres only to a surface denuded of epithelium. When desquamative salpingitis has destroyed the tubal epithelium, the ovum may be impregnated in the tube, and may adhere to the exposed tissue and the dreadful issue of tubal pregnancy will result."

Mr. Tait claims that ovulation is going on constantly, long antecedent to puberty, and, in old age, long after the climacteric. "For pregnancy not only an ovum is required, but it is necessary that the ovum when fertilized should pass over the endometrium when it is denuded of its epithelium, and in a condition of turgescence fit for the subsequent process. But for one ovum thus secured there are probably scores or hundreds which persist, either dropping into the peritoneal cavity or passing out through the uterus. The menstrual process is necessary, or, at least, such parts of it as involve the preparation of the endometrium are necessary for impregnation, but menstruation has nothing to do with ovulation further than being a means to the end of gestation."

Many curious clinical experiences and pathological facts, Mr. Tait argues, are explained by these views. Chronic endometritis, which involves sterility in a large number of cases, is cited. The curette cures the disease and removes the obstruction.

"The condition of infantile uterus means absolute sterility by reason of the arrest in development of the glandular tissue." Mr. Tait further argues that "The singular discrepancy between the uterus and ovaries in their pathological tendencies is due to two facts: the first discovered by Ritchie, that from infancy to death the glandular function of the ovary is never quite at rest, so that ovarian tumors are met with at all ages; the second, displayed for the first time by Johnstone, that the truly glandular function of the uterus begins with puberty and ends with the climacteric, therefore we have myoma practically limited — indeed, in origin the limitation is absolute — to the time between these two incidents of woman's life."

Dr. Johnstone claims in support of his theory that the endometrium above the internal os is not a mucous membrane, but belongs to the so-called adenoid tissues, and that menstruation is for it exactly what the lymph stream is to the lymph gland, or the blood-current to the spleen.

The explanation for the occurrence of this phenomenon in women only, and in some of the higher apes in a partial way, is thus explained: "In two of the ruminants I have shown that nature has supplied this tissue with an abundant lymph-stream, which in the unimpregnated state washes away the ripe material to the general circulation exactly as it does any other lymph corpuscle. But in woman, where, on account of its erect position, the uterus has to depend on the tonicity of its own fibres for the preservation of its shape, no such thing as loose tissue of the lymphatic net-work can be depended on. So, to preserve the integrity of the uterine wall, the emulgent stream is poured into the cavity of the body and got rid of by the vagina."

Dr. Johnstone claims that the sow does not menstruate for the same reason that a child does not. The corpuscles are so slightly developed that they do not need rapid removal.

The theory here advanced is extremely plausible, and, so far as proof has been offered, rests upon well-observed facts. According to this view of the phenomenon of menstruation, the function has been determined by the upright position.—*Maryland Medical Journal.*

